# Probiotic Strain *Lactobacillus casei* BL23 Prevents Colitis-Associated Colorectal Cancer

**DOI:** 10.3389/fimmu.2017.01553

**Published:** 2017-11-17

**Authors:** Elsa Jacouton, Florian Chain, Harry Sokol, Philippe Langella, Luis G. Bermúdez-Humarán

**Affiliations:** ^1^Micalis Institute, INRA, AgroParisTech, Université Paris-Saclay, Jouy-en-Josas, France; ^2^Sorbonne Universités, UPMC Univ. Paris 06, École normale supérieure, CNRS, INSERM, APHP Laboratoire des Biomolécules (LBM), Paris, France

**Keywords:** *Lactobacillus casei* BL23, lactic acid bacteria, probiotic, azoxymethane-dextran sodium sulfate, colorectal cancer, immunomodulation

## Abstract

The gut microbiota plays a major role in intestinal health, and an imbalance in its composition can lead to chronic gut inflammation and a predisposition to developing colorectal cancer (CRC). Currently, the use of probiotic bacteria represents an emerging alternative to treat and prevent cancer. Moreover, consumption of these beneficial bacteria may also favorably modulate the composition of the gut microbiota, which has been described in several studies to play an important role in CRC carcinogenesis. In this context, the aim of this study was to assess the protective effect of oral treatment with *Lactobacillus casei* BL23, a probiotic strain well known for its anti-inflammatory and anticancer properties. First, CRC was induced in C57BL6 mice by a single intraperitoneal injection with azoxymethane (8 mg/kg), followed by four courses of dextran sodium sulfate (2.5%) in drinking water that were separated by an adjustable recovery period. At the time of sacrifice (day 46), tumor incidence, histological scores, and epithelial proliferation were determined in colon samples. Our results show that *L. casei* BL23 significantly protected mice against CRC development; specifically, *L. casei* BL23 treatment reduced histological scores and proliferative index values. In addition, our analysis revealed that *L. casei* BL23 had an immunomodulatory effect, mediated through the downregulation of the IL-22 cytokine, and an antiproliferative effect, mediated through the upregulation of *caspase-7, caspase-9*, and *Bik*. Finally, *L. casei* BL23 treatment tended to counterbalance CRC-induced dysbiosis in mice, as demonstrated by an analysis of fecal microbiota. Altogether our results demonstrate the high potential of *L. casei* BL23 for the development of new, probiotic-based strategies to fight CRC.

## Introduction

Colorectal cancer (CRC) is a major public health problem and is considered the third most common cancer around the world, with nearly 1.2 million new cases every year and a mortality rate of ~40% (1). The incidence of CRC can be associated with a large number of both genetic (2) and environmental factors (3). In particular, one major risk factor for the development of CRC is chronic intestinal inflammation (4); indeed, patients suffering from inflammatory bowel diseases (IBDs) are six times more likely to develop CRC than healthy individuals (5).

Today, the use of probiotics represents a promising strategy for the treatment and prevention of cancer. Probiotics are “live microorganisms, which when administered in adequate amounts confer a health benefit on the host” (6). The most common probiotic strains belong to the genera *Bifidobacterium* and *Lactobacillus*. Interestingly, epidemiological studies have shown a lower incidence of CRC in healthy volunteers who regularly consumed fermented dairy products (containing probiotics), especially yogurt (7–10). However, despite encouraging observations of the anticancer effects of probiotics (which have been accumulating for over 30 years), these clues have thus far been poorly investigated, and even today, the mechanisms underlying these beneficial effects are largely unknown. The beneficial role of probiotic bacteria against CRC onset may be explained by three different mechanisms: (i) modulation of the immune response, (ii) induction of cell apoptosis, or (iii) antioxidant activity [reviewed in Ref. (11)]. As a specific example, the food supplement VSL#3 (a mixture of eight probiotic bacteria) has been shown to modulate the immune response and reduce adenoma development in a model of CRC induced by azoxymethane (AOM) and dextran sodium sulfate (DSS) (12). Furthermore, *Lactobacillus gasseri* and *Bifidobacterium longum* have been shown to inhibit cellular proliferation and increase phagocytic activity in a model of 1,2-dimethylhydrazine (DMH)-associated CRC and thus reduce the multiplicity of aberrant crypt foci as well as tumor size (13). In addition, the probiotic strain *Lactobacillus casei* Shirota is able to suppress chemically induced intradermal tumor onset through both an enhancement of the cytotoxicity of natural killer (NK) cells (14) and IL-12 release by dendritic cells (15).

Beyond having cancer fighting effects, the consumption of probiotics may also favorably modulate the composition of the gut microbiota (16). In this context, several studies have confirmed the important role that the gut microbiota plays in CRC carcinogenesis by generating both biochemical and physiological conditions that may increase the number of colonic preneoplastic lesions (17, 18). However, despite the well-known role of microbiota dysbiosis in CRC pathogenesis, there are conflicting reports about a specific correlation between the bacterial community composition in the gut and susceptibility to CRC (18–20). To date, animal studies have revealed that germ-free mice are more predisposed to developing inflammatory-induced CRC than conventional mice (21). Therefore, manipulation of the gut microbiota could be a promising approach to prevent and/or treat CRC.

We have previously reported anti-inflammatory effects of the probiotic strain *L. casei* BL23 in two different murine models of chemically induced colitis (22–24). Furthermore, we have recently observed that this strain also displays antitumoral properties in a mouse allograft model of human papilloma virus (HPV)-induced cancer and in a DMH-induced CRC model (25). We attributed this antitumoral effect to a modulation of the immune response and, in particular, to the modulation of regulatory T-cells toward a Th17-biased immune response associated with the expression of regulatory cytokines (IL-6, IL-17, IL-10, and TGF-β) (25). However, the beneficial role of *L. casei* BL23 in colitis-associated CRC (such as that induced by AOM-DSS) remains to be assessed.

Thus, keeping in mind the role of the gut microbiota and chronic intestinal inflammation in CRC carcinogenesis, we decided in this study to further investigate the impact of *L. casei* BL23 in a mouse model of CRC induced by AOM and DSS. Our results showed that oral administration of *L. casei* BL23 significantly reduced tumor onset. Histological analyses revealed reduced proliferation in tumor sections, as demonstrated by the upregulation of three genes involved in apoptosis (caspase 7, 9, and Bik) and cytokine IL-22. Illumina sequencing revealed that the gut microbiota of mice treated with *L. casei* BL23 tended to differ in both community composition and community richness from those of PBS-treated mice. Finally, we provide some clues about the host molecular mechanisms involved in the anticancer effects of this beneficial probiotic bacterium.

## Materials and Methods

### Bacterial Strains and Growth Conditions

*Lactobacillus casei* BL23 (26) was grown in Man, Rogosa, and Sharpe medium at 37°C without agitation overnight. To prepare live bacterial inoculum, strains were washed twice with PBS at 3,000 *g* and suspended in PBS at a concentration of 5 × 10^9^ colony-forming units/ml plus 15% glycerol.

### Animals and Experimental Design

All experiments were handled in accordance with institutional ethical guidelines, and the study was approved by the COMETHEA ethics committee (“Comité d’Ethique en Expérimentation Animale”) of the Centre INRA of Jouy-en-Josas and AgroParisTech. Female C57BL/6 mice (6–8 weeks old; Janvier SAS, St. Berthevin, France) were maintained in sterile isolators at the INRA animal facility (*n* = 5 per cage) with 12 h light cycles and fed irradiated normal chow (R 03-40, SAFE) and water *ad libitum*.

**Figure 1:**
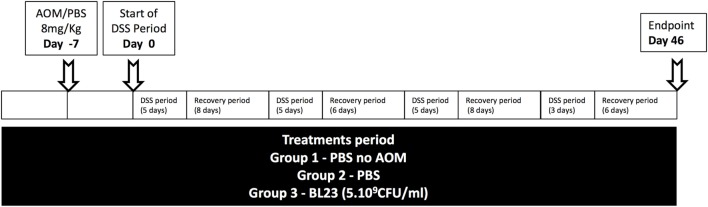
Experimental protocol. AOM, azoxymethane; CFU, colony-forming unit; DSS, dextran sodium sulfate.

Temperature and moisture were carefully controlled. Mice were separated into three groups. The first two groups were administered PBS, while the last group was orally administered *L. casei* BL23. As shown in Figure 1, administration of PBS or *L. casei* BL23 started a week before tumor induction and was performed every day until sacrifice.

Tumors were induced with a single intraperitoneal injection of 8 mg/kg AOM (Sigma-Aldrich) 6 days before the start of a DSS treatment (with the exception of group 1, the negative control). Mice received a 5-day course of 2.5% DSS (TdB) in sterile drinking water, followed by an adjustable recovery period. This schema was followed four times (Figure 1). Intestinal inflammation was assessed daily by measuring the Disease Activity Index (DAI), which included body weight loss (0 = <1%, 1 = 5% > *X* > 1%, 2 = 10% > *X* > 5%, 3 = 15% > *X* > 10%, 4 = > 15%); mouse activity (0 = normal, 2 = hooked back, and 4 = lethargy); stool consistency (0 = absence, 2 = soft and sticky, and 4 = diarrhea); occult/gross rectal bleeding (0 = normal, 1 = occult+, 2 = occult++, 3 = occult+++, and 4 = gross bleeding); and mouse coat state (0 = normal, 2 = ruffed, and 4 = very ruffed). Mice were sacrificed at day 46. Colons were harvested and cleaned with PBS, and tumors were measured with a caliper. Colon sections were stored under conditions appropriate to the subsequent analyses (detailed in the following sections). For each mouse, three sections (~1 cm) were recovered from rectum samples and used for histology, gene expression analysis, and protein analysis. Colic contents were frozen in liquid nitrogen.

### Cytokine Analysis

Mononuclear cells were isolated from spleens by gentle extrusion of the tissue through a 50-μm-mesh Nylon cell strainer (BD). Cells were resuspended in DMEM medium that was supplemented with 10% fetal calf serum, 2 mM l-glutamine, 50 U/mg penicillin, and 50 U/mg streptomycin (Lonza, Levallois-Perret, France). Erythrocytes were lysed with red blood cell lysing buffer (Sigma-Aldrich).

For stimulation experiments, 2.5 × 10^6^ cells per well were cultured for 48 h (37°C, 10% CO_2_) in DMEM medium in P24 plates that were precoated with anti-CD3/CD28 antibodies (4 µg/ml each; eBioscience). Culture supernatant was frozen at −80°C until processing.

Proteins from each colon were extracted with T-PER tissue protein extraction reagent (ThermoFisher Scientific) using a Fastprep instrument at 4,500 *g* for 30 s (two cycles). Samples were centrifuged at 500 *g* for 1 min, and supernatants were harvested for cytokine analysis. A cytometric bead array system (LEGENDplex Mouse Th Cytokine Panel, Biolegend) was used, according to manufacturer’s instructions, to determine the levels of the following cytokines: IL-2, IL-4, IL-5, IL-6, IL-9, IL-10, IL-13, IL-17A, IL-17F, IL-21, IL-22, IFN-γ, and TNF-α.

### Tumor Histology

Colon sections were formalin fixed and embedded in paraffin (4%, VWR, France). Epithelial proliferation was assessed by Ki67 staining according to the manufacturer’s instructions, using mouse monoclonal anti-Ki67 antibody (MM1, Leica Biosystems; 1:50). The proliferation index was determined by counting the number of Ki67-positive cells per crypt in three well-aligned crypts.

Two-micron colon sections were used for H&E staining. Histological score was determined using a BX43 Olympus microscope in a blinded manner, *via* the observation of three parameters: inflammation/cellular infiltration, epithelial lesions, and regeneration.

### Gene and Protein Expression Analysis

Colon sections were stored in RNA later (Ambion) at −80°C. RNAs were extracted using the RNeasy mini-kit (Qiagen, Courtaboeuf, France) following the manufacturer’s recommendations. RNA concentration was measured using a NanoDrop spectrophotometer (NanoDrop Technologies, Wilmington, DE, USA). cDNA synthesis was carried out from 1 µg of RNA using the High Capacity cDNA Reverse Transcription kit (Applied Biosystems, USA), according to the manufacturer’s instructions. RT-qPCR was carried out in a reaction volume of 25 µl with Taqman probes (β-*actin*: Mm01963702_S1, *caspase-9*: Mm00516563_m1, *caspase-7*: Mm01195085_m1, *Bik*: Mm00476123_m1) (Life Technologies, France) according to the manufacturer’s instructions, using an ABI Prism 7700 (Applied Biosystems, USA) thermal cycler. To quantify and normalize the expression data, we used the ΔCt method, using the geometric mean Ct value from β-actin as the endogenous reference gene.

Total proteins were extracted from colon sections using T-PER buffer (Thermoscientific) and protease inhibitor mixture (Roche, Germany), through mechanical lysis with the Precellys homogenizer (Ozyme, France; 2 runs of 4,500 *g* for 30 s). Supernatants were collected, and western blots were performed. Briefly, samples were separated on a mini-PROTEAN TGX precast stain-free gel (4–20%, Biorad). Blots (Trans Blot Turbo Transfer system, Biorad) were incubated with β-actin, caspase-7, and cleaved caspase-7 antibodies (Cell Signaling, Danvers, MA, USA).

### Microbial DNA Extraction

DNA was extracted using the Godon technique from stool (T0 = day of AOM treatment) and cecal content (Tf = time final; day of sacrifice) as described by Lamas et al. (27). The DNA pellet was washed with 70% ethanol, dried, and resuspended in 50 µl of Tris–EDTA buffer. DNA suspensions were stored at −20°C until amplification.

### 16S rDNA Amplification and Gene Analysis

16S rDNA was amplified with primers for the V3 and V4 hypervariable regions (PCR1F_460: 5′-CTTTCCCTACACGACGCTCTTCCGATCTACGGRAGGCAGCAG-3′, PCR1R_460:5′-GGAGTTCAGACGTGTGCTCTTCCGATCTTACCAGGGTATCTAATCCT-3′). The reaction mixture contained 10 ng of genomic DNA, 5 U/μl MTP Taq DNA polymerase (Sigma, France), 0.2 mM dNTP, and 0.5 µM (final concentration) of each primer. Reactions were performed using an annealing temperature of 65°C for 30 cycles in a T100 thermocycler (Biorad, France). Sequencing was performed using 460-bp paired-end reads and an Illumina Miseq protocol on the GeT-PLaGe platform (Toulouse, France). Illumina reads were joined using the fastq-join method. The sequences were demultiplexed and quality filtered using the QIIME (version 1.8.0) software package. The sequences were assigned to OTUs using UCLUST algorithm 41 with a 97% threshold of pairwise identity and classified taxonomically using the Greengenes reference database.

### Statistical Analysis

Data were analyzed with Prism software (version 5). All normally distributed data were displayed as mean ± SEM. Comparisons between two groups were performed with a Student’s *t*-test.

## Results

### *L. casei* BL23 Protects against Tumor Development in a Colitis-Associated CRC Model

**Figure 2:**
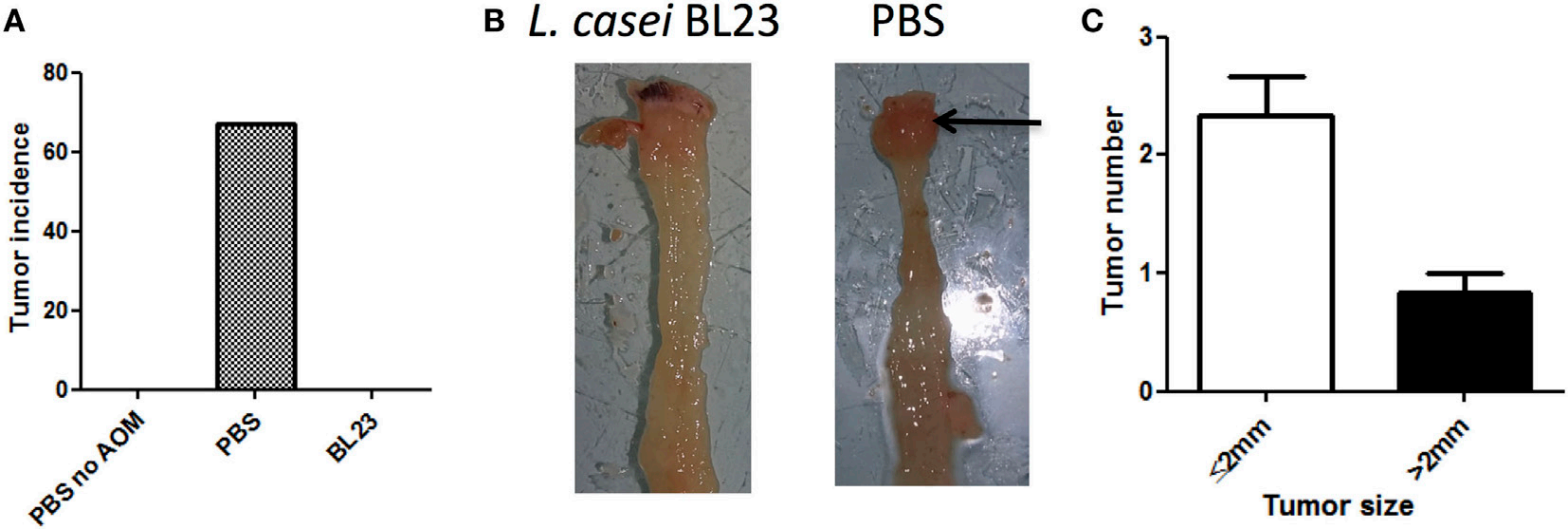
*Lactobacillus casei* BL23 protected against tumor formation. **(A)** Macroscopic colic tumor incidence. Data are represented as the mean of each group ± SEM (*n* = 9 mice) in an *in vivo* experiment. **(B)** Representative view of tumor in a PBS/azoxymethane (AOM)/dextran sodium sulfate (DSS)-treated mouse. **(C)** Colic tumor size in PBS/AOM-treated mice (number of small tumors ≤2 mm and large tumors >2 mm per mouse) in the whole colon. Data are represented as the mean of each group ± SEM (*n* = 9 mice).

**Figure 3:**
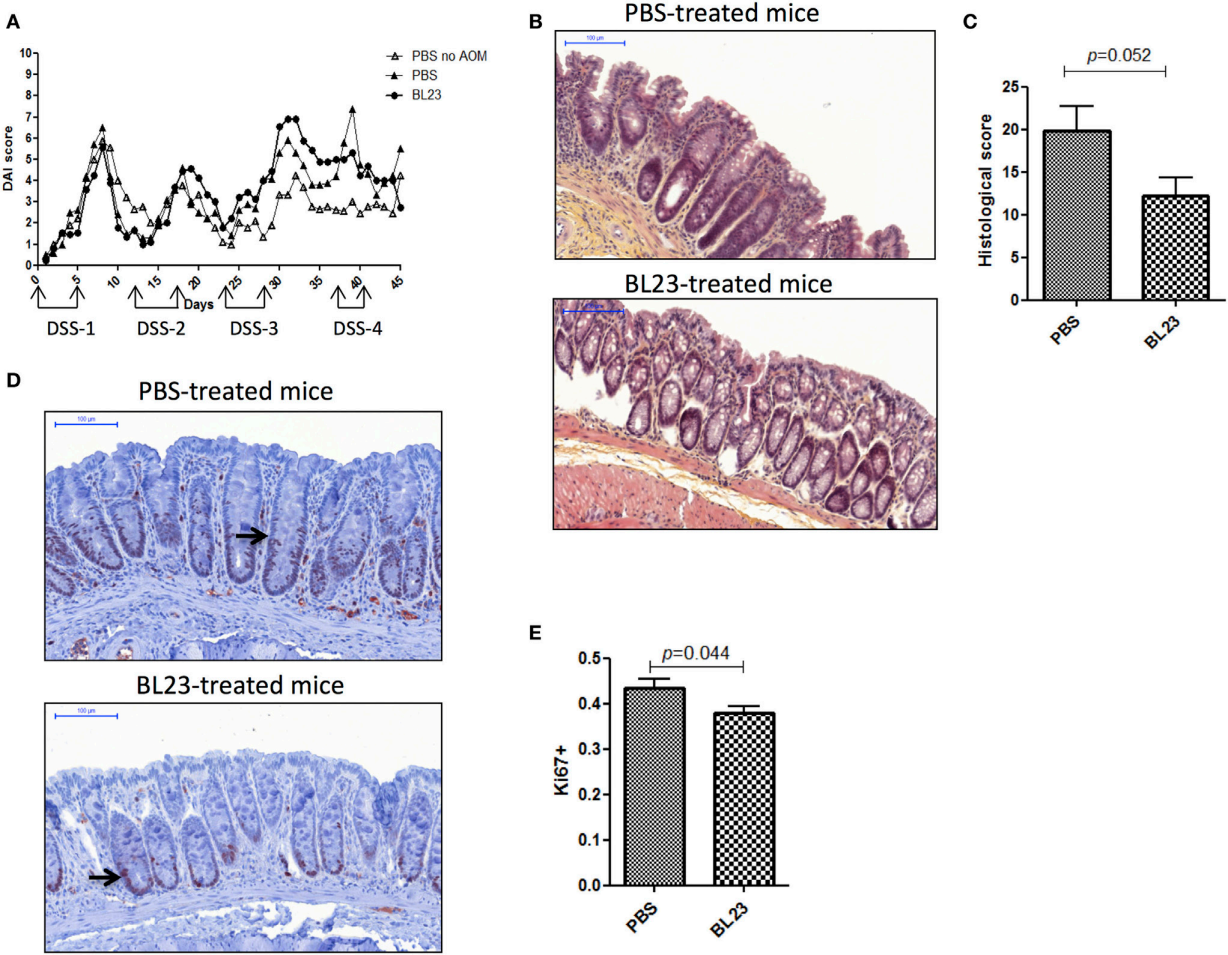
Macroscopic and histological assessment of inflammation and proliferation in mice. **(A)** Disease Activity Index score [dextran sodium sulfate (DSS) treatment periods are indicated on the graph]. **(B)** Representative H&E-stained images of colic tissues from either PBS- or BL23-treated mice at sacrifice; scale bars, 100 µm. **(C)** Semiquantitative scoring of histopathology (*p* = 0.044, Student’s *t-*test). **(D)** Representative Ki67-stained images from either PBS- or BL23-treated mice at sacrifice; scale bars, 100 µm; black arrow indicates Ki67 distribution inside the crypt. **(E)** Proliferative assessment (*p* = 0.052, Student’s *t*-test). Data are represented as the mean of each group ± SEM (*n* = 9 mice) for each graph. AOM, azoxymethane.

To determine the potential beneficial effects of the dairy strain BL23 of *L. casei* on CRC onset, live bacteria were orally administered to mice treated with AOM and DSS. As shown in Figure 2A, mice fed with *L. casei* BL23 were protected against tumor development: no mouse in this group developed macroscopic tumors, compared to 67% (6/9) of mice that received only PBS (Figures 2A,B). Mice treated with PBS developed a per-mouse average of 2.3 tumors at least 2 mm in diameter (Figure 2C). Since this CRC model is related to chronic intestinal inflammation, we then assessed DAI and histological scores and intestinal epithelial damage. As shown in Figure 3A, DAI scores increased after each DSS cycle; however, there were no significant differences between any of the treated groups. For the histological scores, mice fed *L. casei* BL23 showed less damage than control mice did (*p* = 0.052, Student’s *t*-test; Figures 3B,C). In addition, Ki67 levels (which are expressed in proliferating cells) were significantly lower (*p* = 0.044, Student’s *t*-test) in mice treated with *L. casei* BL23 than in control mice (Figures 3D,E).

### *L. casei* BL23 Displays Antiproliferative Activities

**Figure 4:**
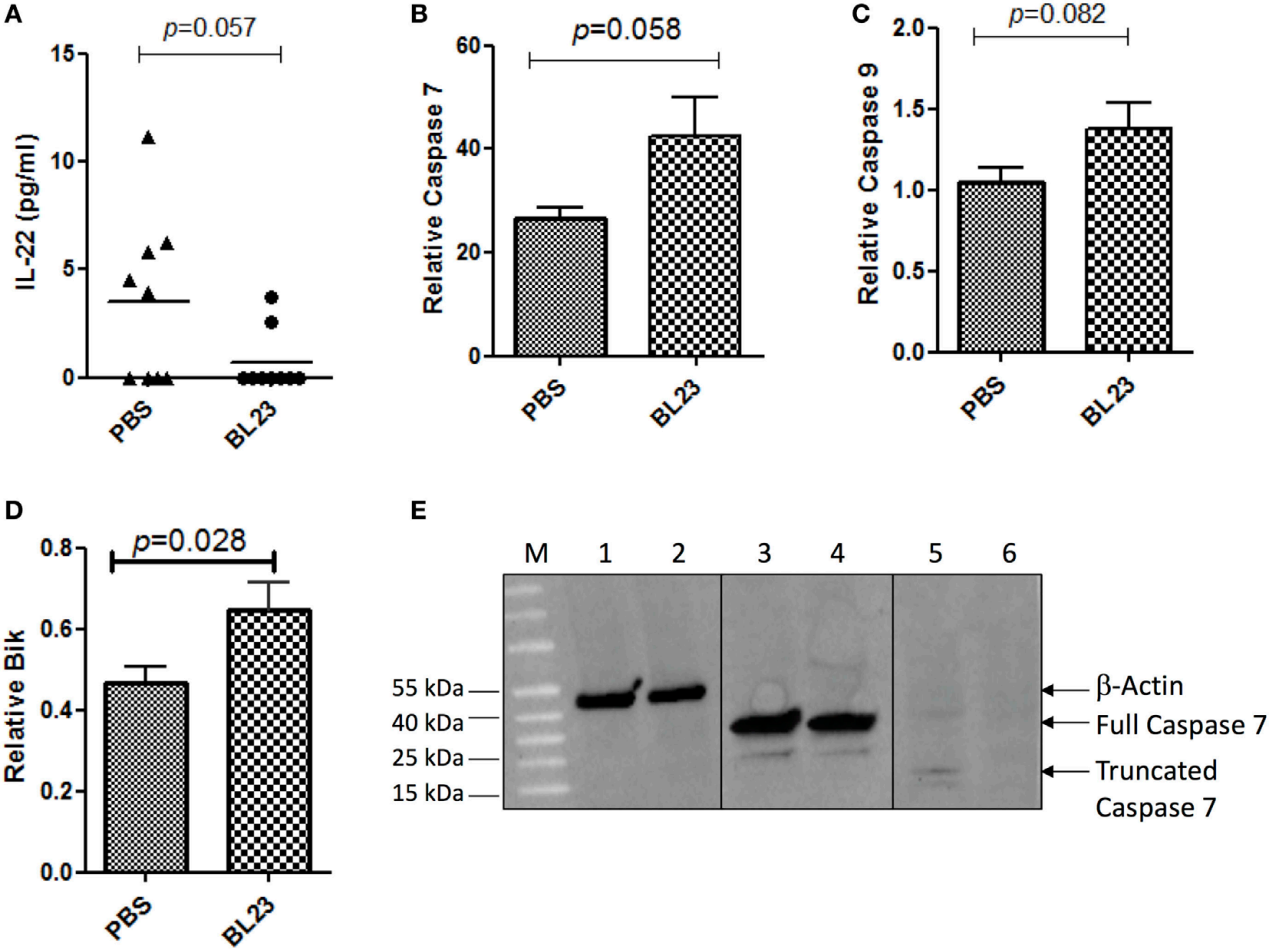
*Lactobacillus casei* BL23 induced antiproliferative activity and increased apoptosis. **(A)** Protein analysis of IL-22 in colon tumor section. **(B)** Real-time PCR analysis of relative expression in colic tumor sections of mRNA of *caspase-7* (*p* = 0.058, Student’s *t*-test), **(C)** *caspase-9* (*p* = 0.082, Student’s *t*-test), and **(D)** *Bik* (*p* = 0.028, Student’s *t*-test). Data are represented as the mean ± SEM of each group (*n* = 9 mice) for each graph. **(E)** Caspase 7 protein expression. Lines 1, 3, and 5 correspond to mice treated with *L. casei* BL23 (and protected against tumors and that expressed high levels of *caspase-7* RNA) and lines 2, 4, and 6 to mice treated with PBS (not protected against tumors). Lines 1 and 2 correspond to samples treated with anti-β-actin antibodies; 3 and 4 with full caspase 7 antibodies; and 5 and 6 with antibodies recognizing the cleaved form of caspase 7.

As immunomodulation and the induction of cell apoptosis are among the main probiotic-related protective mechanisms against CRC, we examined changes in both the immune response and apoptosis pathways due to *L. casei* BL23 treatment. We first determined the levels of both local (i.e., colon and mesenteric lymphoid node (MLN) samples) and systemic (i.e., spleen samples) cytokines that are involved in inflammation and carcinogenesis, including IL-22, IFN-γ, IL-10, IL-21, TNF-α, IL-6, and IL-17A (Figures S1–S3 in Supplementary Material). As shown in Figure 4A, colonic IL-22 (a cytokine that promotes proliferation of cancer cells) (28) levels were lower (*p* = 0.057, Student’s *t*-test) in *L. casei* BL23-treated mice compared to controls. Other cytokines that have been linked to CRC carcinogenesis, such as IFN- γ, TNF-α, IL-6, IL-10, IL-21, and IL-17A, were assessed in MLN (Figure S1 in Supplementary Material), in spleen (Figure S2 in Supplementary Material), and in colon samples (Figure S3 in Supplementary Material), but no significant difference was observed between treated and control mice.

In addition, we performed colic gene expression analysis of genes involved in apoptosis, specifically, *caspase-7, caspase-9*, and *Bik*. Our results reveal that *L. casei* BL23 induced a significant increase compared to controls in the expression of the executioner *caspase-7* (Figure 4B, *p* = 0.017, Student’s *t*-test) and the initiator *caspase-9* (Figure 4C, *p* = 0.028, Student’s *t*-test), together with an increase of the apoptotic gene *Bik* (Figure 4D, *p* = 0.082, Student’s *t*-test). Finally, we determined the level of truncated caspase-7 (which corresponds to the active form of caspase-7 in apoptosis). For this, we selected two mice treated with *L. casei* BL23 and protected against tumor development, which presented high *caspase-7* RNA expression levels, and a mouse treated with PBS (not protected against tumors), which presented low levels of *caspase-7* RNA expression. As shown in Figure 4E, western blot results confirmed that the mice protected against tumor onset produced higher levels of the active form of caspase-7 compared to the non-protected mouse.

**Figure 5:**
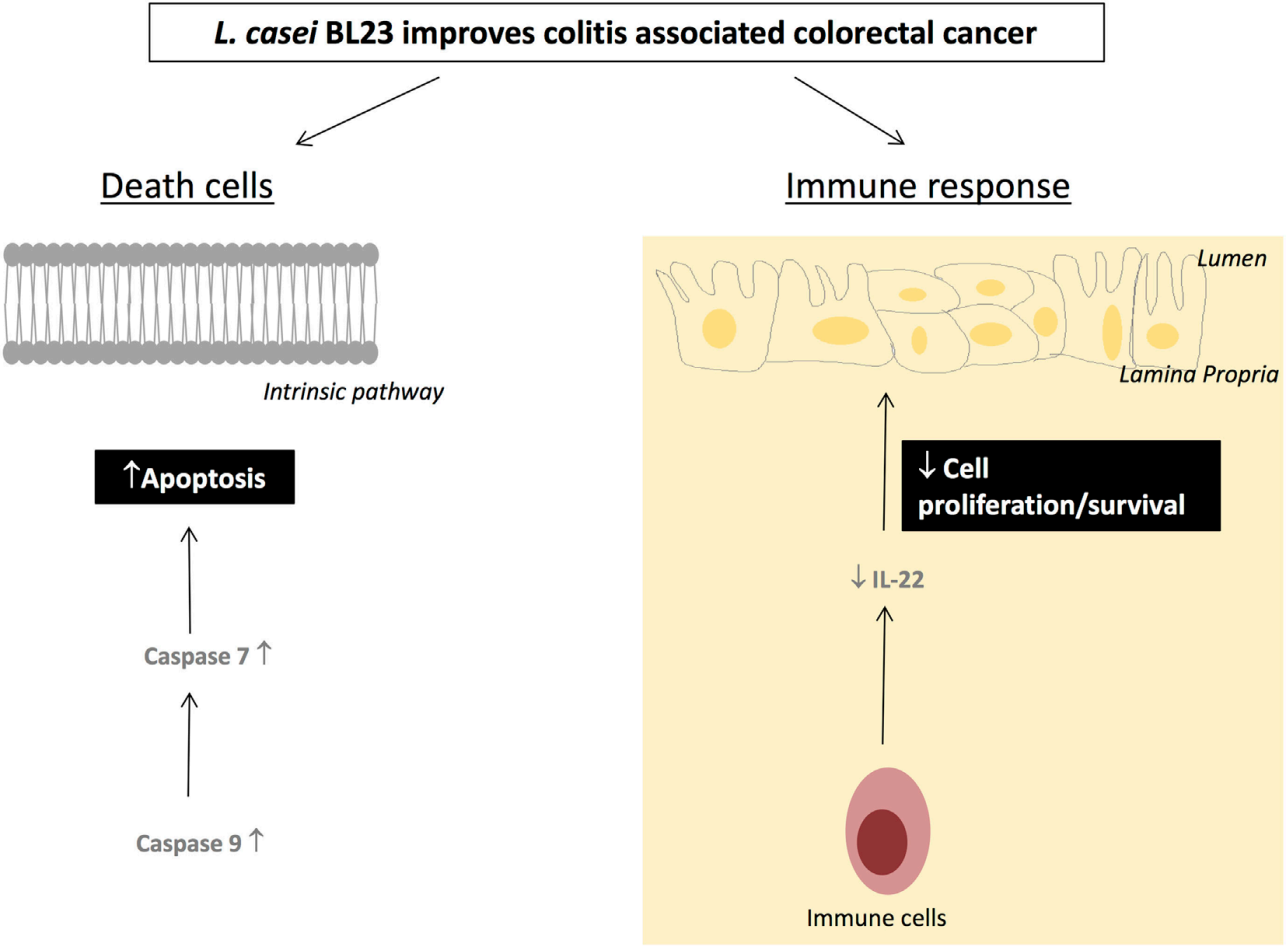
Proposed mechanisms by which *Lactobacillus casei* BL23 protects against tumors.

Altogether, these data suggest that *L. casei* BL23 has an antiproliferative and apoptotic effect in this CRC model; a detailed proposal of the mechanisms of action of *L. casei* BL23 against CRC is shown in Figure 5.

### Impact of *L. casei* BL23 Treatment on Microbiota Richness and Diversity

**Figure 6:**
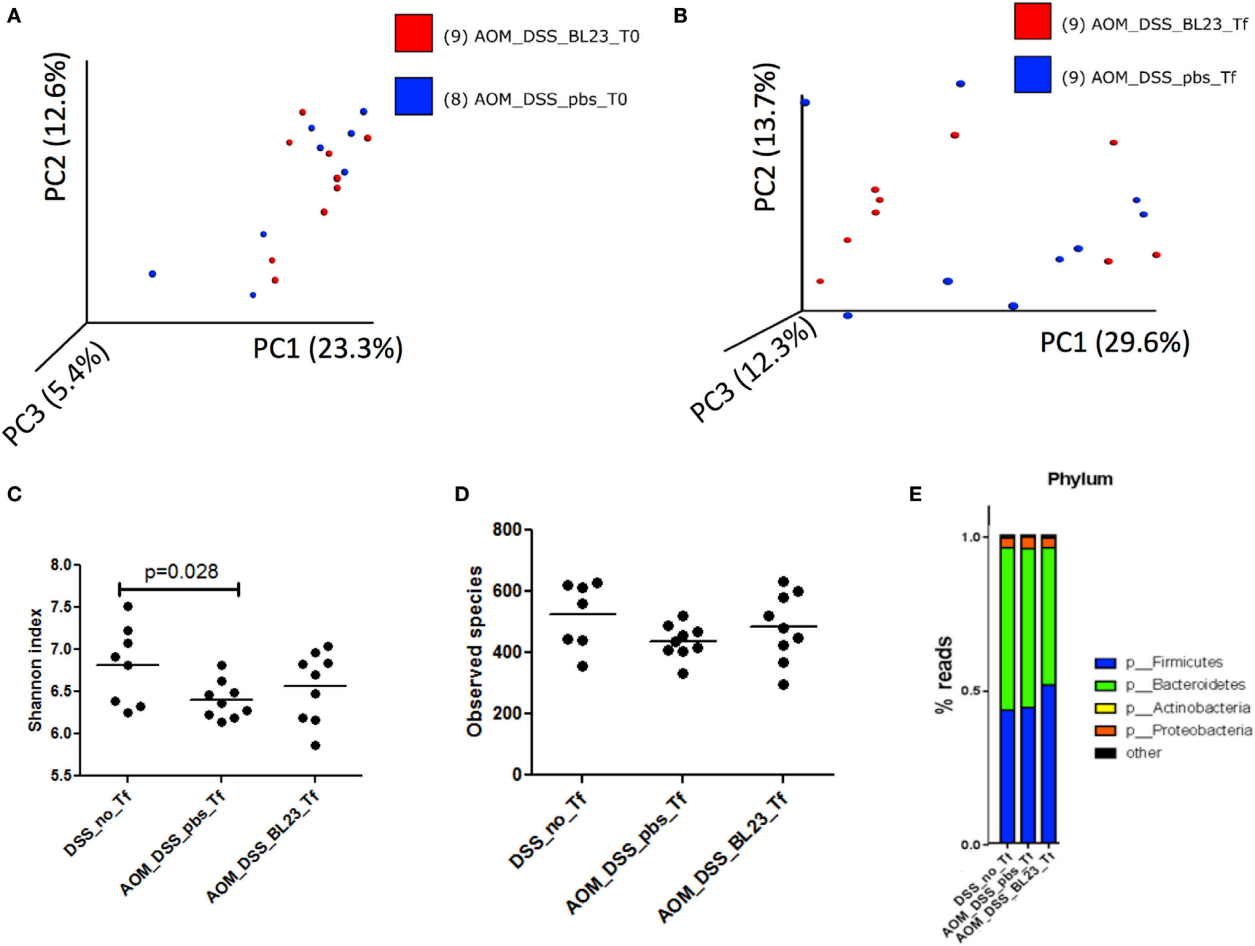
Microbiota analysis. **(A)** Principal components analysis (PCA) of samples at T0. **(B)** PCA at Tf. **(C)** Phylum representation at T0 (*p* = 0.028, Student’s *t*-test). **(D)** Phylum representation at Tf. **(E)** Upregulated and downregulated taxa in BL23 group.

To determine the impact of *L. casei* BL23 on the gut microbiota, we analyzed microbiota richness and diversity after BL23 oral treatment. This analysis provided a total of 1,709,762 high-quality and classifiable reads, with an average of 5,000 (*n* = 51) reads per sample. First, beta diversity (Bray-Curtis distance) was analyzed in microbial samples using principal components analysis, which reduced the dimensionality of the data set. While no grouping was observed before AOM challenge (T0; Figure 6A), the microbiota of mice treated with *L. casei* BL23 tended to diverge from those of PBS-treated animals at Tf (sacrifice; Figure 6B, *p* = 0.1, Anosim). Then, we estimated community richness (alpha diversity; Shannon and Simpson indexes) at T0 and Tf. As shown in Figure 6C, AOM injection resulted in a reduction in alpha diversity with respect to that found in the group treated with PBS but not AOM (Shannon index, 6.4 ± 0.22 in PBS/AOM-treated group versus 6.8 ± 0.46 in PBS/no AOM group, *p* = 0.028, Student’s *t*-test). Despite the fact that the effects of *L. casei* strain BL23 were not statistically significant (Simpson index, 483.3 ± 111.8 in BL23/AOM-treated group *versus* 436.8 ± 54.7 in PBS/AOM-treated group, ns, Student’s *t*-test; Figure 6D), these results provide intriguing clues about this strain’s interactions with other members of the gut microbiota.

Finally, bacterial communities were characterized at the phylum level (Figure 6E). Firmicutes was the dominant phylum in the *L. casei* BL23/AOM treated group (51 ± 10%), with Bacteroidetes ranked second (45 ± 10%). In contrast, Bacteroidetes was the most abundant phylum in both PBS/AOM and PBS/no AOM treated groups (52 ± 12 and 53 ± 13%, respectively), followed by Firmicutes (44 ± 12 and 43 ± 13%, respectively). The third most abundant phylum in all groups was Proteobacteria, with approximately 3–4% of reads. By using linear discriminant analysis (LDA) coupled with effect size measurements (linear discriminant analysis effect size), we found that *Prevotella*, Ruminococcaceae, and *Lactobacillus* were the key groups that were overrepresented in the *L. casei* BL23-treated mice (Table 1). At the species level, only *Lactobacillus zeae* was significantly more abundant (*p* = 0.004, Student’s *t*-test) in these mice, with a LDA score of 2.58. This was not surprising, since this species could in fact correspond to strain BL23 of *L. casei*. Indeed, 16S RNA analysis has revealed a close relationship (99% similarity) between our focal strain and *L. zeae* (26).

**Table 1:** Linear discriminant analysis effect size in BL23-treated group at Tf.

Taxa	LDA score	*p* Value
Bacteroidetes. Bacteroidia. Bacteroidales. Paraprevotellaceae. Prevotella	3.2891683	0.038
Firmicutes. Clostridia. Clostridiales. Ruminococcaceae	3.01945662	0.012
Firmicutes. Bacilli. Lactobacillales. Lactobacillaceae. *Lactobacillus zeae*	2.58044456	0.004
Tenericutes	2.573562	0.040
Tenericutes. Mollicutes	2.57347147	0.040
Actinobacteria. Actinobacteria. Bifidobacteriales. Bifidobarteriaceae. Bifidobacterium	−0.44318815	0.004
Firmicutes. Clostridia. Clostridiales	−0.43837615	0.011
Actinobacteria. Actinobacteria	−0.42251683	0.018
Actinobacteria. Actinobacteria. Bifidobacteriales. Bifidobacteriaceae. Bifidobacterium	−0.40710059	0.029
Firmicutes. Clostridia. Clostridiales. Clostridiaceae. Clostridium	−0.38186026	0.030
Proteobacteria. Alphaproteobacteria	−0.367064	0.019

## Discussion

There is now mounting evidence pointing to an important link between both chronic colic and rectal damage (present, for example, in IBD patients) and CRC carcinogenesis. Indeed, the risk of developing colitis-associated cancer increases by ~1% in IBD patients (5). To better understand the mechanisms related to tumor onset, different preclinical animal models of CRC have been developed, such as the AOM-DSS model used in this study (29).

*L. casei* BL23 has been previously studied for its anti-inflammatory activities in different models of chemically induced colitis (22, 23). Furthermore, *L. casei* has been widely studied in different murine models of cancer (14, 30–32). In particular, *L. casei* BL23 has antiproliferative effects in the mouse allograft model of HPV-induced cancer and protects against DMH-induced CRC (25). Here, we decided to explore the impact of oral administration of *L. casei* strain BL23 in a murine model of CRC induced by AOM and DSS. Strikingly, our results revealed that *L. casei* BL23 significantly reduced tumor development, since all treated mice were tumor free at the end of the experiment. In addition, we also found some clues about the molecular mechanisms involved in the protective effect against cancer, and it appears that, in this model, *L. casei* BL23 acts mainly *via* the inhibition of cell proliferation. Indeed, this strain is able to downregulate proliferation, as observed through a decrease in Ki67. In addition, *L. casei* BL23 was also able to increase apoptosis *via* upregulation of *caspase-9, caspase-*7, and *Bik*.

Given the reported anti-inflammatory properties of *L. casei* BL23, we also assessed the cytokine profiles of treated mice. With the exception of a reduced histological score, *L. casei* BL23 had no significant effects on cytokine regulation. However, a weak decrease was observed in IL-22 levels in colons from mice treated with BL23. This cytokine has been recently implicated in CRC development in both humans and APC^min/+^ murine model (33). Thus, it appears that IL-22 levels are enhanced in tumor tissues and that mice displaying lower levels of this cytokine are protected from tumorigenesis. It was recently reported that the beneficial effects of a strain of *Lactobacillus reuteri* in a model of CRC induced by AOM-DSS were mediated by a histidine decarboxylase (HDC), which downregulated IL-22 expression (34). We performed *in silico* analyses to search for the nucleotide sequence of the HDC cluster in the *L. casei* BL23 genome, but were unable to find a corresponding sequence region (data not shown). However, it is still possible that another protein produced by this strain may act directly on IL-22 regulation/expression. The main sources of IL-22 are NK cells, γδ T cells, and lymphoid tissue inducer cells, as well as TH17 and TH22 cells. To determine which cell types are affected by *L. casei* BL23 treatment, future experiments will need to examine the correlation between each population of cells in the lamina propria of mice and IL-22 downregulation.

Finally, several reports have described disruption of the composition of the microbiota in CRC (17, 18). Therefore, we analyzed the bacterial diversity (16S rDNA) in fecal samples from our mice and found that *L. casei* BL23 tended to restore the diversity disrupted by AOM injection. In agreement with previous reports (20), Firmicutes was the dominant phylum in the AOM/PBS-treated group. However, the introduction of *L. casei* BL23 reversed the ratio of Firmicutes to Bacteroidetes. Few individual species were affected by *L. casei* treatment, with the exception of *L. zeae*, which is actually considered a synonym of *L. casei* strain BL23 (26). However, future investigations should also consider analyzing the bacterial communities in the colic mucosa from tumor sections, because the assemblages present in stools are not necessarily an accurate reflection of the intestinal environment.

In conclusion, our work revealed that *L. casei* strain BL23 protected against CRC in an AOM/DSS model. Although this bacterium has well-known anti-inflammatory properties, we speculate instead that the protection observed here occurs through a reduction in cell proliferation and the induction of apoptosis.

## Ethics Statement

All experiments were handled in accordance with institutional ethical guidelines, and the study was approved by the COMETHEA ethics committee (“Comité d’Ethique en Expérimentation Animale”) of the Centre INRA of Jouy-en-Josas and AgroParisTech. Female C57BL/6 mice (6–8 weeks old; Janvier SAS, St. Berthevin, France) were maintained in sterile isolators at the INRA animal facility (*n* = 5 per cage) with 12 h light cycles and fed irradiated normal chow (R 03-40, SAFE) and water *ad libitum*.

## Author Contributions

EJ, LB-H, and FC conceived and designed the study. EJ and HS performed data analysis. EJ and LB-H wrote the manuscript. EJ conducted all experiments. FC provided technical help for the *in vivo* experiments. EJ, LB-H, PL, and HS discussed the experiments and results.

## Conflict of Interest Statement

The authors declare that the research was conducted in the absence of any commercial or financial relationships that could be construed as a potential conflict of interest.

## Supplementary Material

Figure S1 Cytokine expression levels in mesenteric lymphoid node. **(A)** IFN- γ, **(B)** IL-17A, **(C)** IL-6, **(D)** TNF-α, **(E)** IL-10, and **(F)** IL-22. Medians are represented for each group.

Figure S2 Cytokine expression levels in spleen. **(A)** IFN-γ, **(B)** IL-17A, **(C)** IL-6, **(D)** IL-22, **(E)** TNF-α, **(F)** IL-10, and **(G)** IL-21. Medians are represented for each group.

Figure S3 Cytokine expression levels in colon. **(A)** IFN-γ, **(B)** IL-17A, **(C)** IL-6, **(D)** TNF-α, **(E)** IL-10, and **(F)** IL-21. Medians are represented for each group.
