# Supplementary figures and images for: Probiotic Strain Lactobacillus casei BL23 Prevents Colitis-Associated Colorectal Cancer
Source: Front Immunol. 2017 Nov 17;8:1553. doi: 10.3389/fimmu.2017.01553 (PMC5702231; doi:10.3389/fimmu.2017.01553)

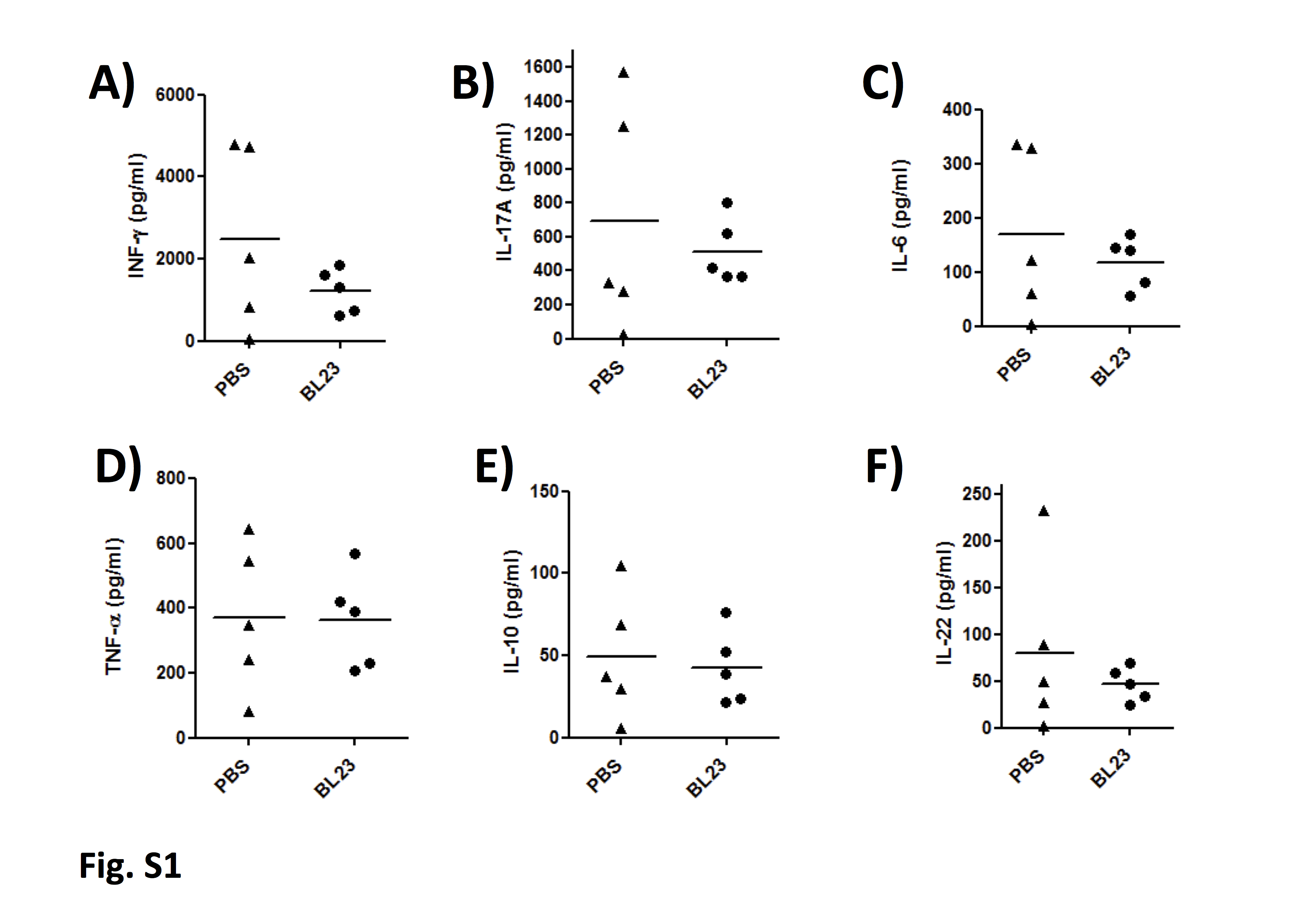

Supplement: Figure S1 — Cytokine expression levels in mesenteric lymphoid node. (A) IFN- γ, (B) IL-17A, (C) IL-6, (D) TNF-α, (E) IL-10, and (F) IL-22. Medians are represented for each group. [file Image_1.tiff]

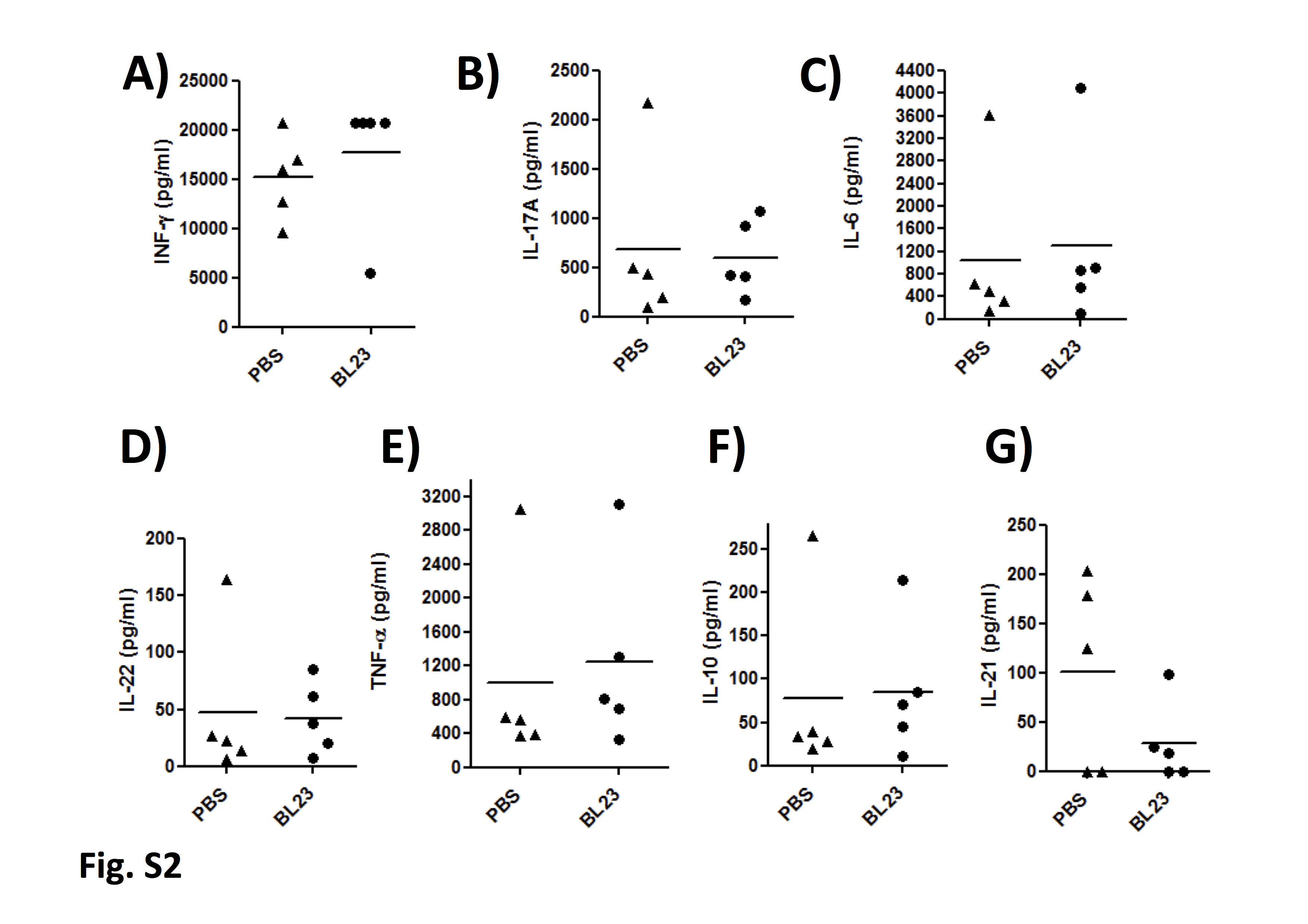

Supplement: Figure S2 — Cytokine expression levels in spleen. (A) IFN-γ, (B) IL-17A, (C) IL-6, (D) IL-22, (E) TNF-α, (F) IL-10, and (G) IL-21. Medians are represented for each group. [file Image_2.tiff]

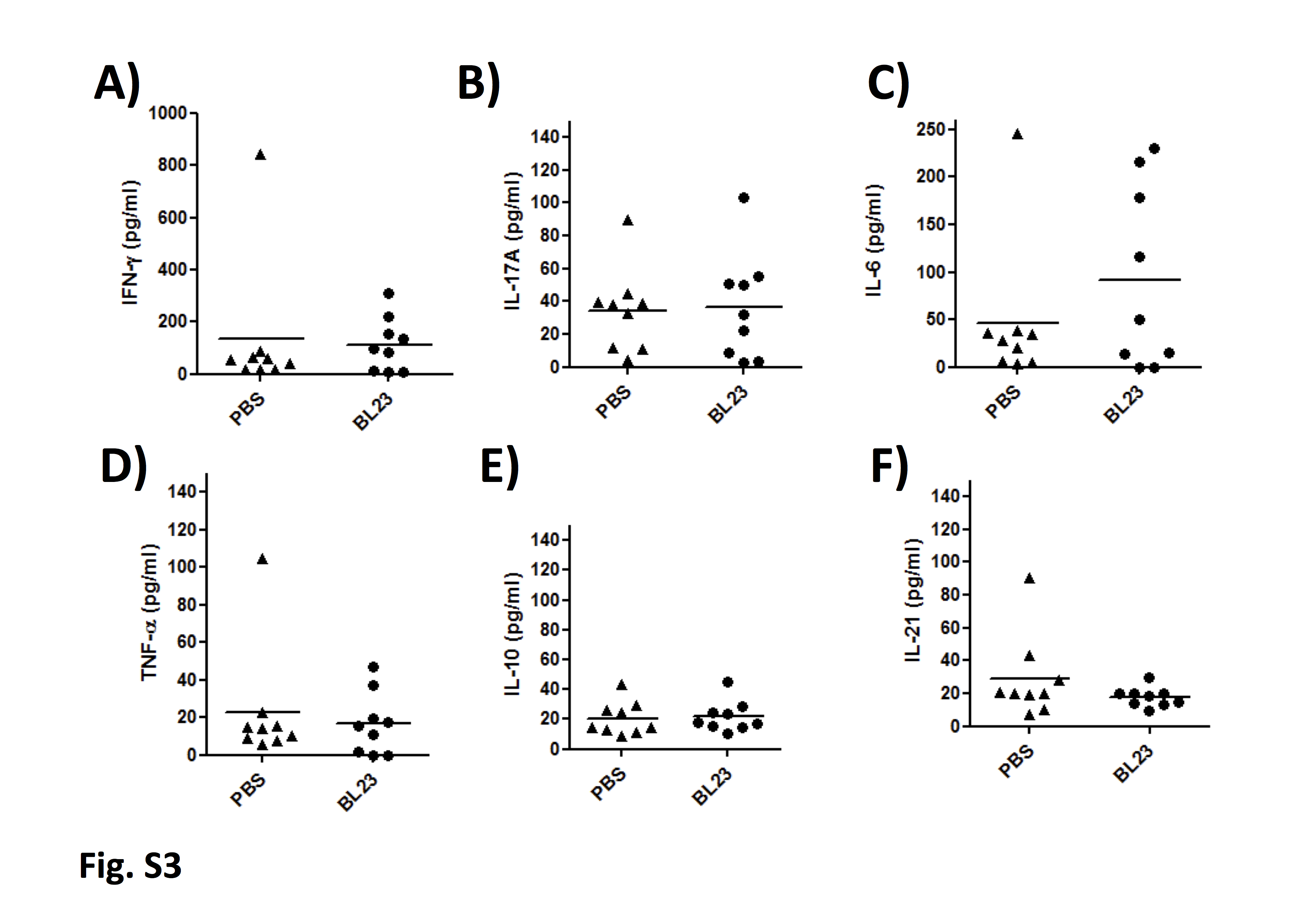

Supplement: Figure S3 — Cytokine expression levels in colon. (A) IFN-γ, (B) IL-17A, (C) IL-6, (D) TNF-α, (E) IL-10, and (F) IL-21. Medians are represented for each group. [file Image_3.tiff]
